# Supplementary material for: Unraveling the Impact of Acetylation Patterns in Chitosan Oligomers on Cu2+ Ion Binding: Insights from DFT Calculations
Source: Int J Mol Sci. 2023 Sep 7;24(18):13792. doi: 10.3390/ijms241813792 (PMC10531048; doi:10.3390/ijms241813792)
Supplement: Supplementary file 1 [file ijms-24-13792-s001.zip › ijms-2550372-supplementary.pdf]

Unraveling the Impact of Acetylation Patterns in Chitosan Oligomers on Cu^{2+} Ion Binding: Insights from DFT Calculations

Ratna Singh [1,*], Jens Smiatek [2] and Bruno M. Moerschbacher [1,*]

[1] Institute for Biology and Biotechnology of Plants, University of Münster, Schlossplatz 8, 48143 Münster, Germany
[2] Institute for Computational Physics, Universität Stuttgart, Allmandring 3, 70569 Stuttgart, Germany; smiatek@icp.uni-stuttgart.de
* Correspondence: singhr@uni-muenster.de (R.S.); moersch@uni-muenster.de (B.M.M.)

Supplementary

Table S1 Distance between Cu^{2+} and 3'O or 2'N in GlcN- Cu^{2+} (M) complexes in bridge and pendant form

coordination method		tetra BLYP Å	penta BLYP Å	hexa BLYP Å
**Cu-3'OH**				
	**bridge**	2.0	2.1	2.3
**Cu-2'NH$_2$**				
**i)**	**bridge**	2.0	2.0	2.0
**ii)**	**pendant**	2.0	2.0	2.0

Table S2 Free energy of complex formation and bond dissociation energy of GlcN- Cu^{2+} (M) complexes

coordination method		tetra BLYP kcal/mol	penta BLYP kcal/mol	hexa BLYP kcal/mol
**GlcN-Cu bridge**				
**i)**	**free energy**	−69.9	−40.7	−20.9
**ii)**	**dissociation energy**	85.7	52.7	30.8
**GlcN-Cu pendant**				
**i)**	**free energy**	−36.7	−23.1	−12.9
**ii)**	**dissociation energy**	52.7	34.1	28.7

Table S3 Distance between Cu^{2+} and 3'O or 2'N in GlcN-Cu^{2+} (M) complexes within chitosan dimers and trimers (D = GlcN; A = GlcNAc)

	2'N-Cu^{2+} (Å)	3'O-Cu^{2+} (Å)
GlcN-(GlcN with Cu^{2+}), (D-M)	2.0	2.1
(GlcN with Cu^{2+})-GlcN, (M-D)	2.0	2.2
(GlcN with Cu^{2+})-GlcNAc, (M-A)	2.0	2.3
GlcNAc-(GlcN with Cu^{2+}), (A-M)	2.0	2.1
GlcN-GlcN-(GlcN with Cu^{2+}), (D-D-M)	2.0	2.1
GlcN-(GlcN with Cu^{2+})-GlcN, (D-M-D)	2.0	2.4
(GlcN with Cu^{2+})-GlcN-GlcN, (M-D-D)	2.0	2.3
GlcNAc-(GlcN with Cu^{2+})-GlcN, (A-M-D)	2.0	2.5
GlcN-(GlcN with Cu^{2+})-GlcNAc, (D-M-A)	2.0	2.2
GlcNAc-(GlcN with Cu^{2+})-GlcNAc, (A-M-A)	2.0	2.4

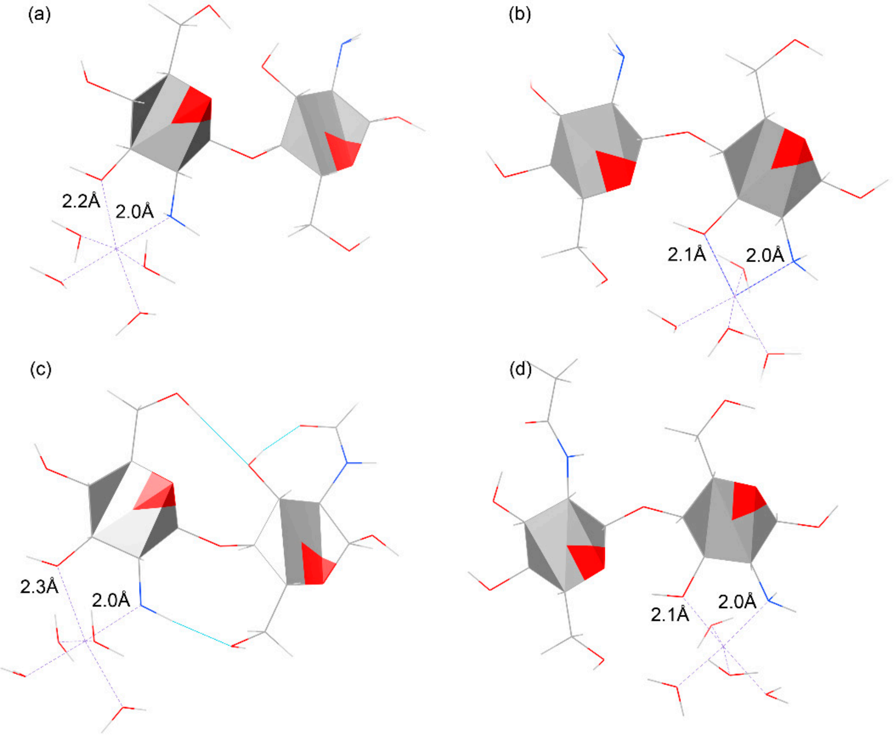

Figure S1 Distance between Cu^{2+} and 3'O or 2'N in GlcN-Cu^{2+} (M) complexes within chitosan dimers after geometry optimization, using the BLYP functional; a) GlcN-GlcN with Cu^{2+} bound to the non-reducing end GlcN (M-D); b) GlcN-GlcN with Cu^{2+} bound to the reducing end GlcN (D-M); c) GlcN-GlcNAc with Cu^{2+} bound to the GlcN (M-A); d) GlcNAc-GlcN with Cu^{2+} bound to the GlcN (A-M). Increasing bond length between Cu^{2+} and 3'O indicates weaker interactions.

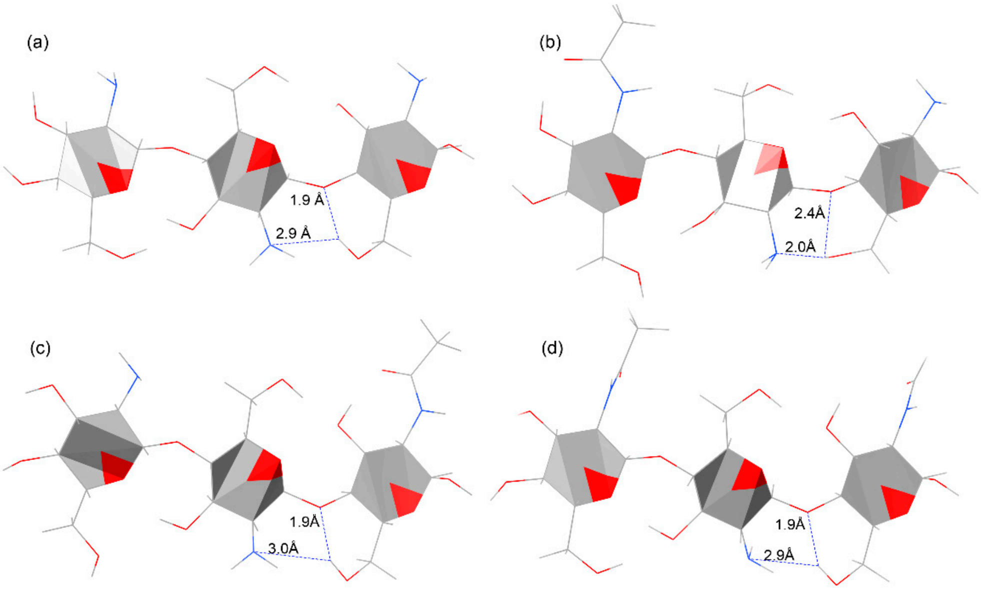

Figure S2 Intramolecular interaction in the chito-oligomers a) GlcN-GlcN-GlcN (D-D-D); b) GlcNAc-GlcN-GlcN (A-D-D); c) GlcN-GlcN-GlcNAc (D-D-A); d) GlcNAc-GlcN-GlcNAc (A-D-A). In ADD, intramolecular interactions and their bond length were mapped differently compared to DDD, DDA and ADA.

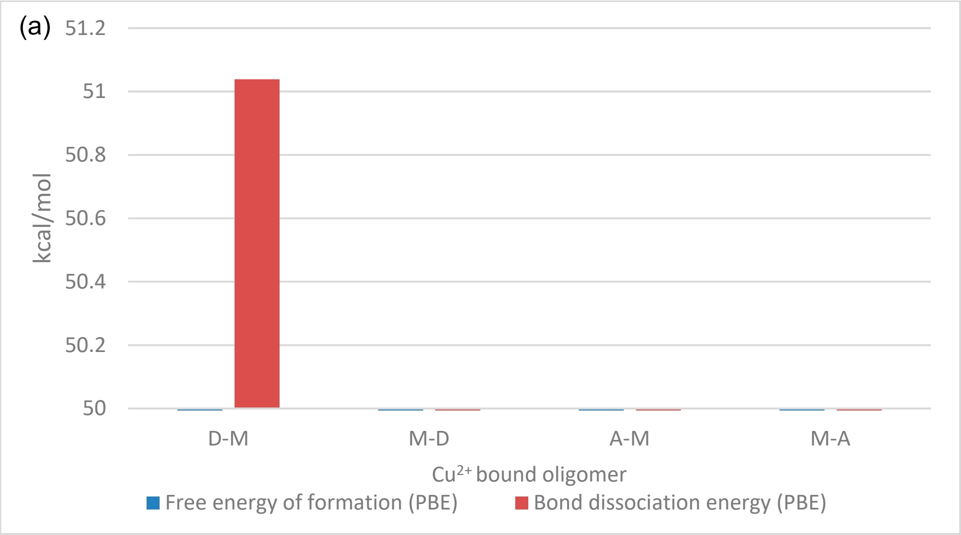

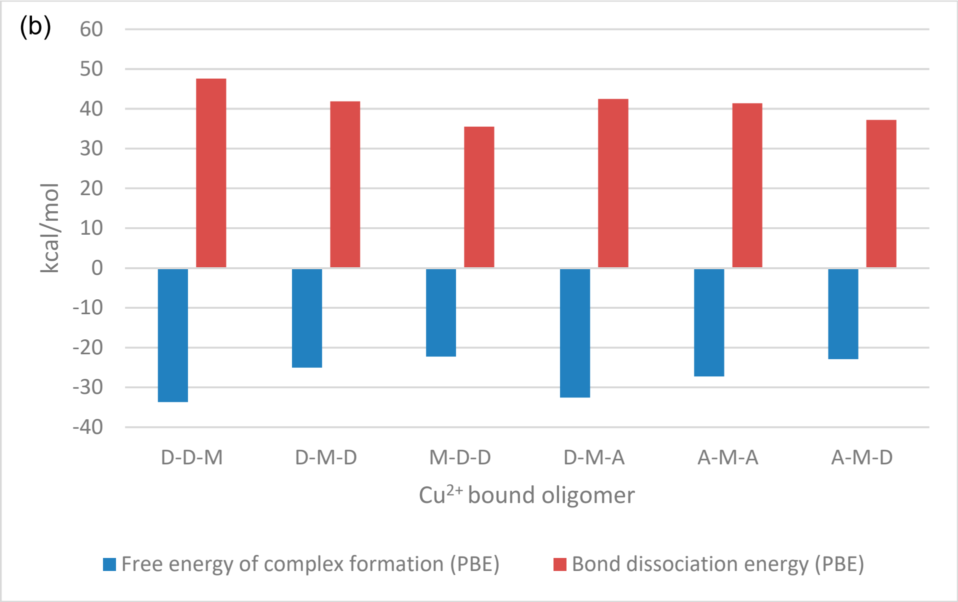

Figure S3 Free energy of copper-chitosan complex formation (a) chitosan dimers (b) selected chitosan trimers calculated using PBE, where M represents the complex of GlcN and Cu^{2+} bound to the 2'NH_2 and 3'OH sites of the same subunit; A = GlcNAc, D = GlcN, M = GlcN-Cu^{2+} complex.

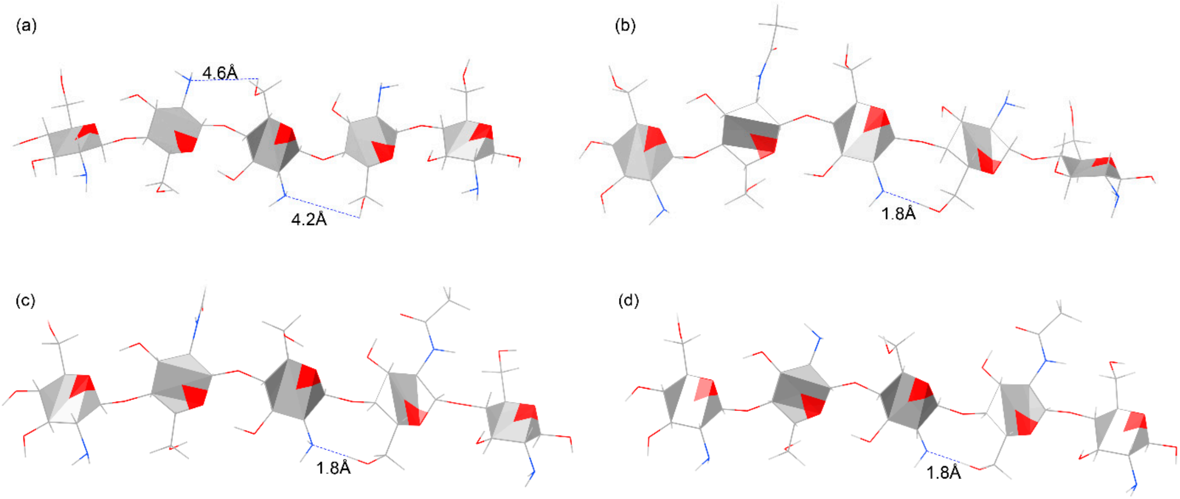

Figure S4 Intramolecular interaction between 2'N and 6'H in neighboring units within chitosan pentamers; a) GlcN-GlcN-GlcN-GlcN-GlcN (D-D-D-D-D); b) GlcN-GlcNAc-GlcN-GlcN-GlcN (D-A-D-D-D); c) GlcN-GlcNAc-GlcN-GlcNAc-GlcN (D-A-D-A-D); d) GlcN-GlcN-GlcN-GlcNAc-GlcN (D-D-D-A-D). New intramolecular interaction in the partially acetylated oligomers indicates the influence of acetylated group on the structure and their polarity.

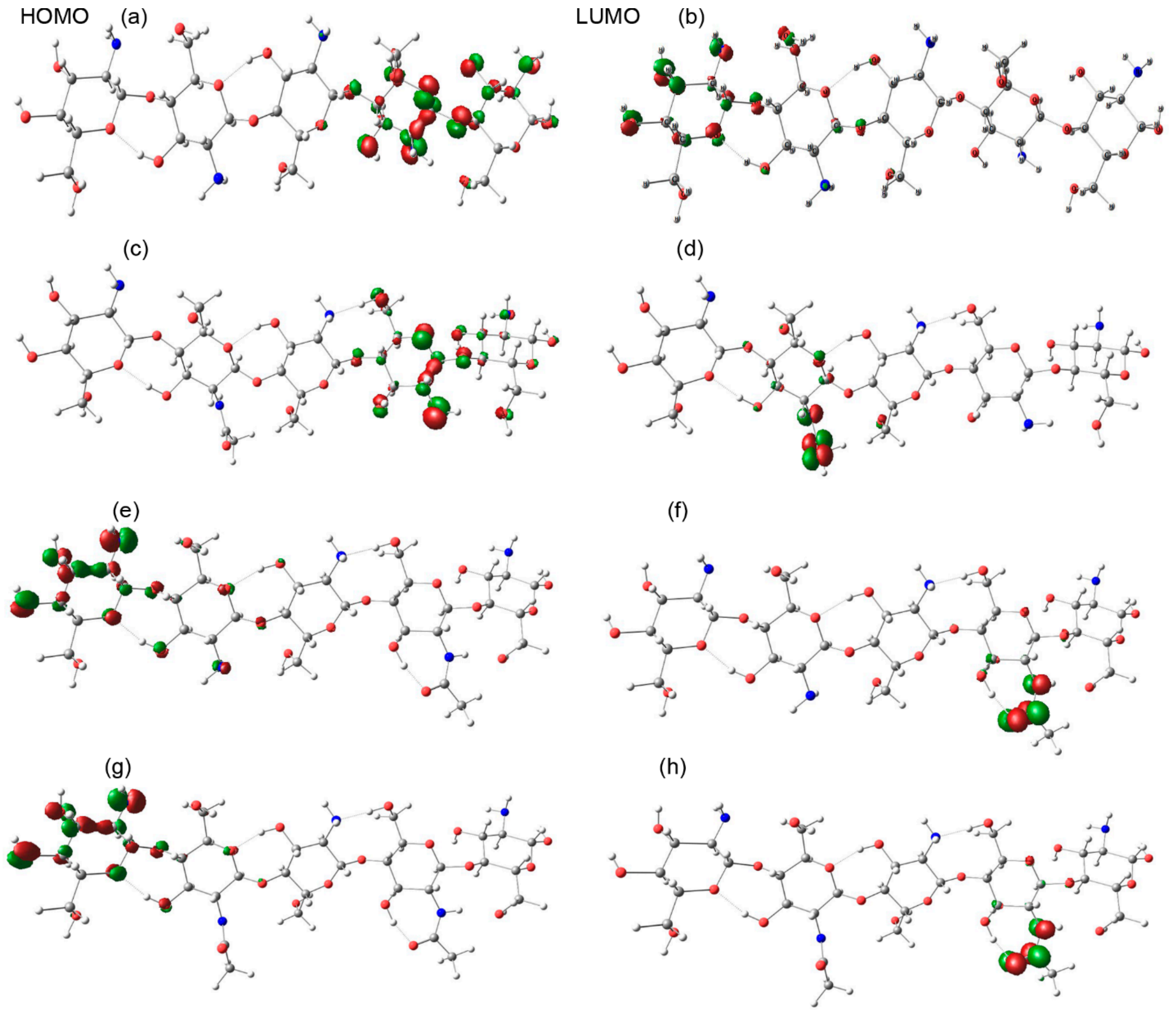

Figure S5 Positions of HOMO and LUMO in chitosan pentamers; a) GlcN-GlcN-GlcN-GlcN-GlcN (D-D-D-D-D), HOMO; b) GlcN-GlcN-GlcN-GlcN-GlcN (D-D-D-D-D), LUMO; c) GlcN-GlcNAc-GlcN-GlcN-GlcN (D-A-D-D-D), HOMO; d) GlcN-GlcNAc-GlcN-GlcN-GlcN (D-A-D-D-D), LUMO; e) GlcN-GlcN-GlcN-GlcNAc-GlcN (D-D-D-A-D), HOMO; f) GlcN-GlcN-GlcN-GlcNAc-GlcN (D-D-D-A-D), LUMO; g) GlcN-GlcNAc-GlcN-GlcNAc-GlcN (D-A-D-A-D), HOMO; h) GlcN-GlcNAc-GlcN-GlcNAc-GlcN (D-A-D-A-D), LUMO.
